# Increase in secreted airway mucins and partial Muc5b STAT6/FoxA2 regulation during *Pneumocystis* primary infection

**DOI:** 10.1038/s41598-019-39079-4

**Published:** 2019-02-14

**Authors:** Diego A. Rojas, Pablo A. Iturra, Andrea Méndez, Carolina A. Ponce, Rebeca Bustamante, Miriam Gallo, Pamela Bórquez, Sergio L. Vargas

**Affiliations:** 1 Biomedical Sciences Institute, University of Chile School of Medicine, Independencia 1027, Independencia, Santiago 8380453 Chile; 2 Servicio Médico Legal de Santiago, Av. La Paz 1012, Independencia, Santiago 8380454 Chile

## Abstract

Airway mucus responses to subclinical infections may explain variations in progression of chronic lung diseases and differences in clinical expression of respiratory infections across individuals. *Pneumocystis* associates to more severe Chronic Obstructive Pulmonary Disease (COPD), asthma, respiratory distress of premature newborns, and is a consistent subclinical infection between 2 and 5 months of age when hospitalizations for respiratory cause and infant mortality are higher. This atypical fungus associates to increased mucin 5AC (MUC5AC), a central effector of Th2-type allergic inflammation, in infant lungs. However, mucus progression, expression of MUC5B essential for airway defense, and potential for pharmacologic modulation of mucus during *Pneumocystis* infection remain unknown. We measured MUC5B and *Pneumocystis* in infant lungs, and progression of mucin levels and effect of inhibition of the STAT6/FoxA2 mucus pathway using Kaempferol, a JAK/STAT6 inhibitor, in immunocompetent rats during *Pneumocystis* primary infection. *Pneumocystis* associated to increased MUC5B in infant lungs. Muc5b increased earlier and more abundantly than Muc5ac during experimental primary infection suggesting an acute defensive response against *Pneumocystis* as described against bacteria, while increased Muc5ac levels supports an ongoing allergic, Th2 lymphocyte-type response during primary *Pneumocystis* infection. Kaempferol partly reversed Muc5b stimulation suggesting limited potential for pharmacological modulation via the STAT6-FoxA2 pathway.

## Introduction

Airway mucus is a biological hydrogel barrier that protects the airway against physical, chemical and biological insults. Healthy mucus is composed by water (>90%) and by polysaccharides and proteins whose relative proportions determine rheological properties and may vary in airway disease impairing mucociliary clearance^1,2^. Changes in mucus composition can importantly alter mucus transport and airway clearance contributing to mucous plugging as in asthma^3,4^. Upregulated mucus is characteristic of chronic diseases like asthma and COPD^2,5,6^.

Mucins are the main structural components of mucus. They consist of high molecular weight proteins classified into gel-forming and tethered mucins. MUC5AC and MUC5B are the main gel-forming mucins. They are secreted and heavily glycosylated^1,2^ contributing to form a very adhesive gel blanket that lies over periciliary fluid and is mechanically propelled by airway cilia to clean the airways. MUC5B is more secreted by submucosal glands and MUC5AC by superficial airway epithelial cells^2,7,8^. Additional mucins, classified as tethered mucins, are associated to the surface of the airway epithelium. Mucus production is tightly controlled via nonspecific mucogenic pathways such as IL13/JAK/STAT6 that controls mucin expression by binding inhibition of transcriptional repressor FoxA2 to mucin promoters^9^, TNFα/NFκβ, IL1β/COX-2, the Gabaergic system, EGFR mediated signaling, and others^5,10–12^. Mucus hypersecretion suggests stimulation of mucogenic pathways^10–12^. MUC5AC secreted by goblet cells in the airway epithelium, is a central effector of allergic inflammation and is required for airway hyperreactivity^7,13^. This mucin was considered for many years the most abundant mucin in the pediatric airways. MUC5B however, has been recognized more recently to be far more abundant than MUC5AC in healthy and asthmatic children^4^ and in adults with pulmonary fibrosis where regulation via FoxA2 promoter binding has been documented^14^. MUC5B has an essential role in defense against bacterial pneumonia, and lack of this mucin severely affected infection-related survival in animal models^8,10^.

*Pneumocystis*, a fungus well-known by the severe pneumonia of immunocompromised individuals, associates to increased severity of Chronic Obstructive Pulmonary Disease (COPD)^15^, to respiratory distress syndrome of newborns^16^, and is likely the most frequent and consistent infection of early infancy^17^. This primary *Pneumocystis* infection of immunocompetent infants goes undetected and peaks between two and five months of age^18–21^ providing a particular epidemiologic context that coincides with the highest prevalence of infant hospitalizations for respiratory cause^22,23^. We have reported increased MUC5AC and CLCA1 associated to *Pneumocystis* primary infection in lungs of infants dying in the community^19,24^. Understanding the effect of *Pneumocystis* on mucin production and its controlling pathways is underscored by the epidemiological context of this fungal infection^18–21^ and by the demonstration that *Pneumocystis* primary infection induces a Th2 environment in the healthy lung^25,26^. MUC5AC has been recently described as an essential effector of the epithelial response to allergic inflammation^7^. Increased MUC5AC is consistent with the intense Th2 (allergic type) airway immune response^25,26^ and STAT6 pathway activation^27^ plus induction of mucus-related genes such as Muc5ac and Clca3^28^ associated to mucus hypersecretion documented in animal models of *Pneumocystis* infection^26,27,29^. Of interest, anti-Muc5ac immune staining is able to recognize only a minimal fraction of the mucus that stains with Alcian blue, suggesting additional mucins are involved during *Pneumocystis* infection^26^. No studies of MUC5B expression in infant lungs or of the murine homolog gene Muc5b during *Pneumocystis* infection are available.

This work shows for the first time, that *Pneumocystis* associates to increased levels of MUC5B in infants, and replicates this finding in an experimental animal model of naturally acquired primary infection that resembles the mode of contagion and course of the primary infection in humans. We also show that pulmonary Muc5b occurs earlier and is more abundant that Muc5ac, that the mechanism of Muc5b hypersecretion partly depends on STAT6 stimulation by *Pneumocystis* -*that inhibits FoxA2 repressor*-, and that this mechanism can be reversed by pharmacological de-repression of FoxA2 using Kaempferol, a specific inhibitor of JAK3 that activates STAT6^30^.

## Results

### MUC5B determinations in infant lungs

**Figure 1:**
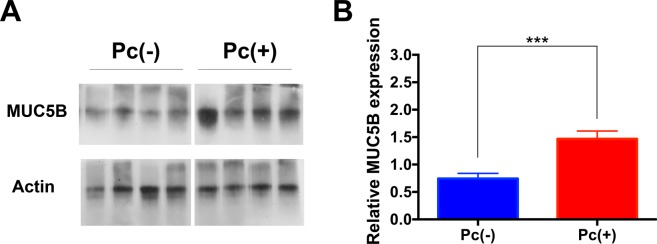
MUC5B determinations in infant lungs. (**A**) Representative MUC5B western blot showing *Pneumocystis* negative (Pc−) and *Pneumocystis* positive (Pc+) infant lung samples. Actin was measured as loading control. (**B**) Graph plot of MUC5B western blot quantification levels. Each bar represents the mean +/− SD of 8 observations normalized by actin. *P*. value was calculated using Mann-Whitney test. ^***^ indicates *P* = 0.0002.

The clinical characteristics of infants whose autopsy lung biopsies were selected for this study were previously described^19^. Eight *Pneumocystis*-positive and eight control samples were randomly selected for analysis from a pool of 39 *Pneumocystis*-positive and 20 *Pneumocystis*-negative lung specimens. MUC5B protein levels increased in *Pneumocystis*-infected compared to non-infected infants (*P* < 0.001) (Fig. 1).

### Muc5b and Muc5ac mRNA and protein levels in lungs of *Pneumocystis* primary infection experimental and control animals

**Figure 2:**
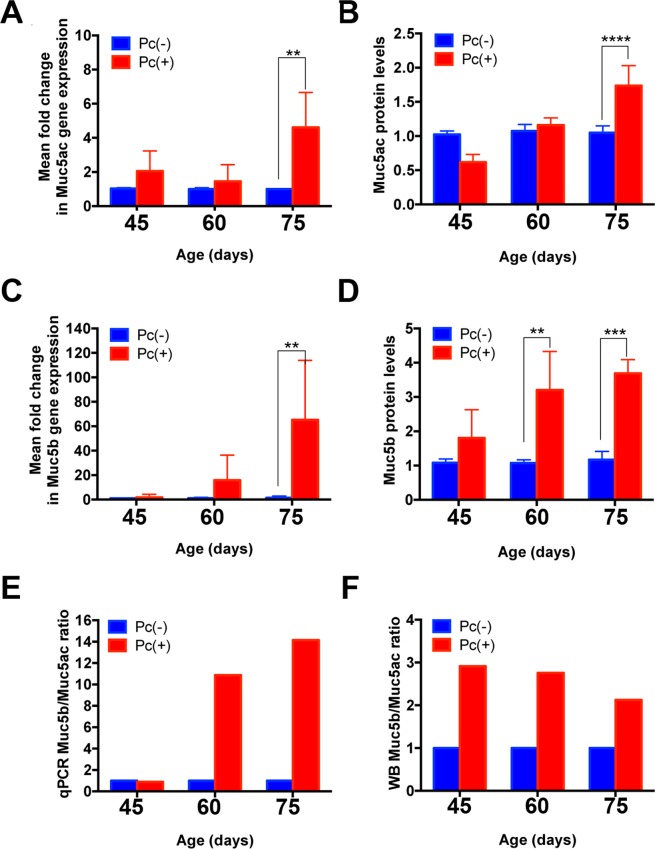
Mucin gene-expression and protein quantifications at 45, 60, and 75 days after *Pneumocystis* co-habitation exposure. Muc5ac mRNA (**A**), and protein levels (**B**) and, Muc5b mRNA (**C**) and protein levels (**D**) were determined by qPCR and Western blot, respectively. The qPCR and WB Muc5b:Muc5ac ratios are shown in (**E** and **F**). Note that scales are different. Each bar represents the mean +/− SD of qRT-PCR determinations calculated using the 2^−ΔΔCt^ method, or the mean +/− SD of the respective lung mucin determination protein levels in 4 rats. Results were normalized by actin and compared with the results of their respective control group. Significance was evaluated by two-way ANOVA and *P* values were A: ^**^P = 0.0017; B: ^****^P = 0.0001; C: ^**^P = 0.0082; D: ^**^P = 0.0014 on day 60 and ^***^P = 0.0002 on day 75. Results are expressed in arbitrary units.

mRNA levels of Muc5b were determined by qRT-PCR and compared with Muc5ac levels at each sacrifice point. mRNA levels of both mucins increased from day 60 and reached significance on day 75 (Fig. 2A,C). Of interest, the mRNA levels of Muc5b in the *Pneumocystis*-positive group increased over 10-fold than those of Muc5ac (Fig. 2E). Immunoblotting showed that protein expression levels of Muc5ac increased on day 75 (Fig. 2B), and Muc5b increased from day 60 reaching significance on days 60 and 75 (Fig. 2D). Muc5b protein levels were over 2-fold higher than those of Muc5ac in *Pneumocystis*-positive animals (Fig. 2F). *Pneumocystis* lung burden in these animals, estimated by qPCR of the Dihydrofolate Reductase (*dhfr*) gene of *Pneumocystis*, was characterized in previously published experiments^26^.

### Immunohistochemistry for Muc5b and Muc5ac

**Figure 3:**
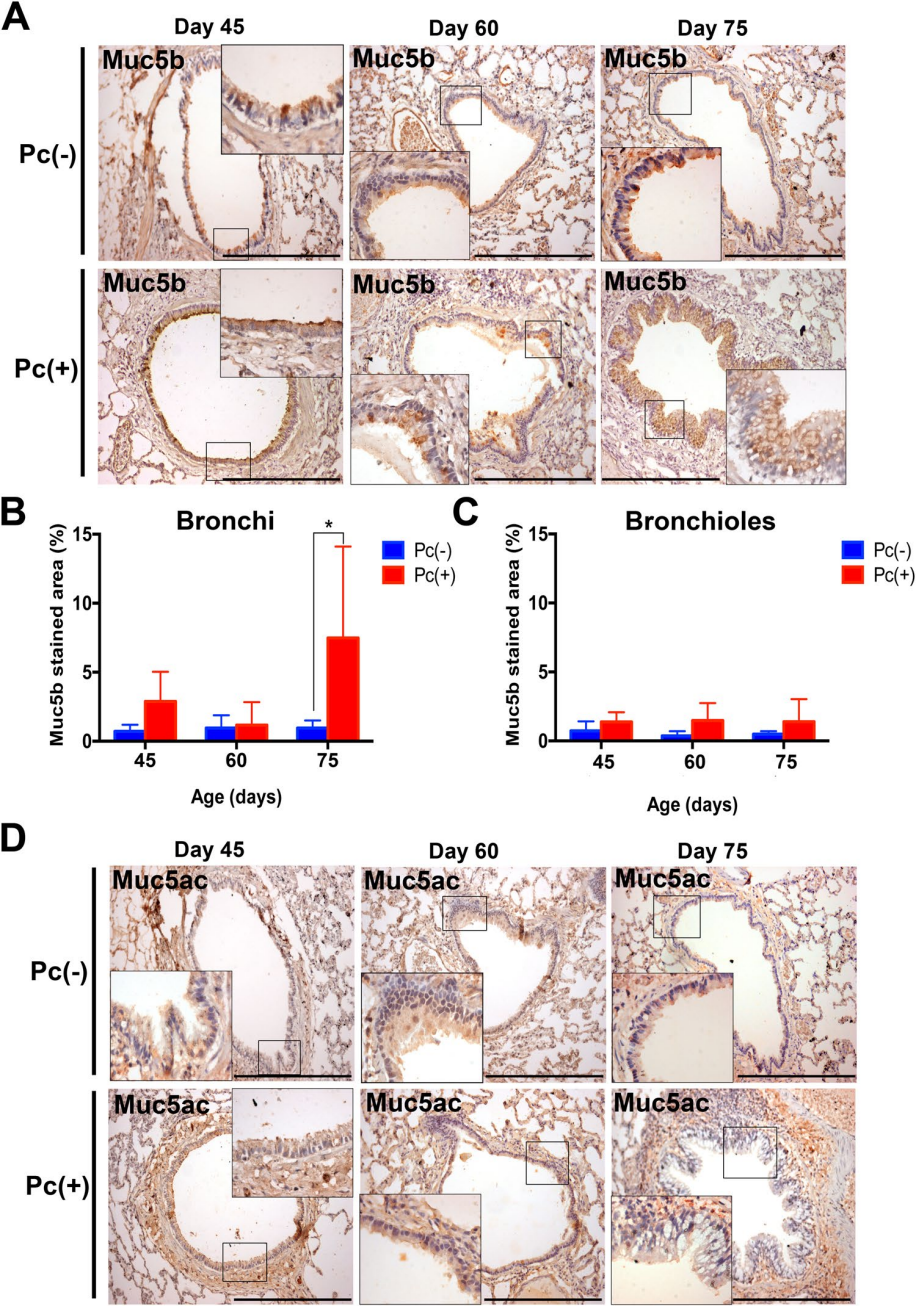
Mucin determinations by immunohistochemistry in bronchi (>250 μm diameter airways) and bronchioles (50–250 μm diameter airways). (**A**) Muc5b: airway epithelium area stained using an anti-Muc5b antibody in lung sections of *Pneumocystis*-positive and negative rats at 45, 60, and 75 days post *Pneumocystis* exposure using an 100X amplification, and 400X insets. Black bar represents 200 μm. (**B**) Graph plot comparison of the percentage of epithelial area of bronchi and bronchioles occupied by Muc5b in *Pneumocystis*-positive and negative rats at 45, 60, and 75 days post *Pneumocystis* exposure. Results are expressed as the mean percentage of DAB-positive area of airway epithelium +/− SD. Statistical comparisons were determined using two-way ANOVA. ^*^ indicates *P* = 0.0335. (**C**) Muc5ac: representative immunohistochemically stained sections. Distribution of Muc5ac stained area was focal and hindered histological quantification. Areas staining positive are shown using an 100X amplification, and 400X insets.

Airways were classified according to diameter in bronchi (>250 μm) and bronchioles (<250 μm)^31^. Terminal bronchioles (<50 μm) were not observed in these microscopy slide sections. Muc5b was highly detected in bronchi of infected animals on days 45 and 75 of age compared to those of controls (Fig. 3A,B). Muc5b determinations on bronchioles of control and infected animals were not different (Fig. 3C). Signal for Muc5ac was not visualized in sections adjacent to those positive for Muc5b (Fig. 3D).

### Effect of Kaempferol, a specific inhibitor of the JAK/STAT6 pathway, on *Pneumocystis*-induced STAT6 phosphorylation

**Figure 4:**
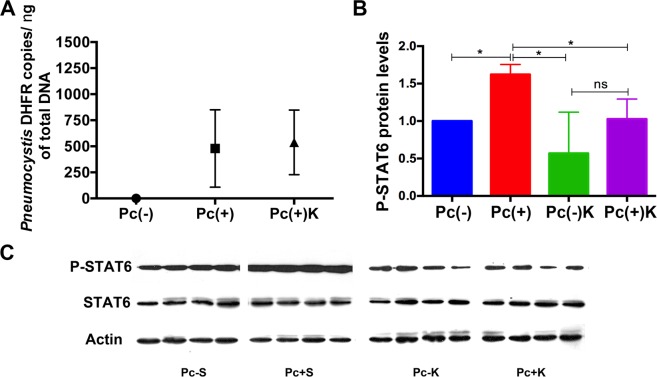
STAT6 pathway activation by *Pneumocystis* and pharmacological reversal with Kaempferol. Kaempferol was administered by intraperitoneal injection from day 55 to 70 of age. (**A**) Pneumocystis burden quantified by qPCR amplification of the *dhfr* gene in DNA from *Pneumocystis*-negative (control), *Pneumocystis*-positive without Kaempferol and *Pneumocystis*-positive with Kaempferol, rat lungs at day 70 of the experiment. (**B**) Graph plot and (**C**) Western Blot show that Kaempferol inhibited the *Pneumocystis* stimulatory effect. Each bar represents the mean ± SD of P-STAT6 protein levels as determined by western blot in lung samples from 4 rats. STAT6 and Actin were evaluated as internal controls. Graph plots were normalized by STAT6 and Actin. Statistical values for comparisons of the means were determined using Mann-Whitney. ^*^ indicates *P* = 0.0286. Pc(−) represents *Pneumocystis* non-infected rats injected with saline, Pc(+) represents *Pneumocystis-infected rats* injected with saline, Pc(−)K *Pneumocystis* non-infected rats injected with Kaempferol and Pc(+)K *Pneumocystis*-infected rats injected with Kaempferol.

The phosphorylation state of transcription factor STAT6 was evaluated in lung tissue using anti-Phospho STAT6 (P-STAT6) specific antibodies and Western Blot. P-STAT6 protein levels were increased in *Pneumocystis*-infected Kaempferol-untreated animals respect to control rats treated with saline. The *Pneumocystis* burden reached similar levels than in lungs where muc5ac^26^ and muc5b mucins were quantitated (Fig. 4A). The P-STAT6 stimulation effect of *Pneumocystis* was attenuated by Kaempferol as documented by the decrease in phosphorylation of STAT6 factor in *Pneumocystis*-infected animals receiving Kaempferol compared to those *Pneumocystis*-infected animals receiving saline (Fig. 4B,C).

### Effect of Kaempferol in *Pneumocystis*-induced Muc5b expression

**Figure 5:**
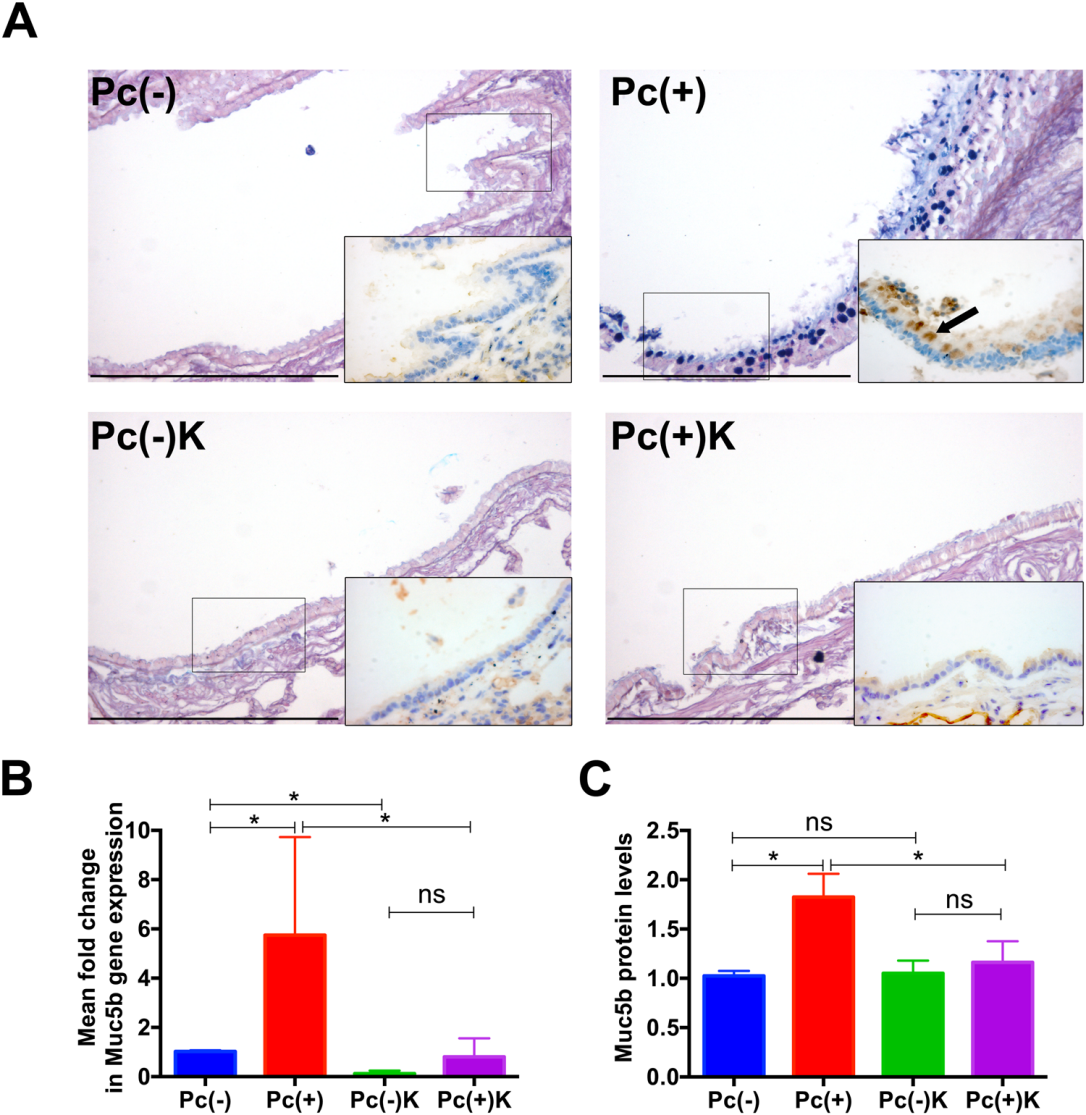
Modulation of MUC5B expression by Kaempferol administration. (**A**) Representative AB/PAS stained microscopy lung sections (100X) with insets (400X) to show immunohistochemically stained MUC5B in airway epithelium. Bar represents 200 μm. (**B**) Graph plot of MUC5B mRNA levels in lung samples from animals of each experimental group. Each bar represents the mean fold change in gene expression of 4 determinations using 2^−ddCt^ method. Data were normalized by actin and compared with the result of the Pc(−) control group. (**C**) MUC5B protein levels as determined by western blot in lung samples from each experimental group. Each bar represents the mean +/− SD of 4 determinations normalized by Actin. Significance between quantifications were determined using Mann-Whitney. ^*^ indicates *P* = 0.0286. *ns* indicates no significance.

Mucus documented by AB/PAS staining and by immunohistochemistry with anti-Muc5b antibody in *Pneumocystis* infected rats receiving saline (Pc+) coincide in airway epithelium location (Fig. 5A & arrow in inset) and was attenuated by administration of Kaempferol (Fig. 5A). Muc5b mRNA levels increased in *Pneumocystis*-infected animals receiving saline and this increase was 6-fold higher when compared with *Pneumocystis* non-infected rats (Fig. 5B). Treatment with Kaempferol completely reversed this effect to the level of *Pneumocystis* non-infected rats receiving saline (Pc−, Fig. 5B). Muc5b protein expression increased 2-fold in the *Pneumocystis* infected group when compared with the non-infected controls (Fig. 5C). The changes induced by Kaempferol in Muc5b protein levels replicated at a lesser scale the Kaempferol effect in mRNA levels.

### Effect of *Pneumocystis* in transcriptional regulation of Muc5b promoter

**Figure 6:**
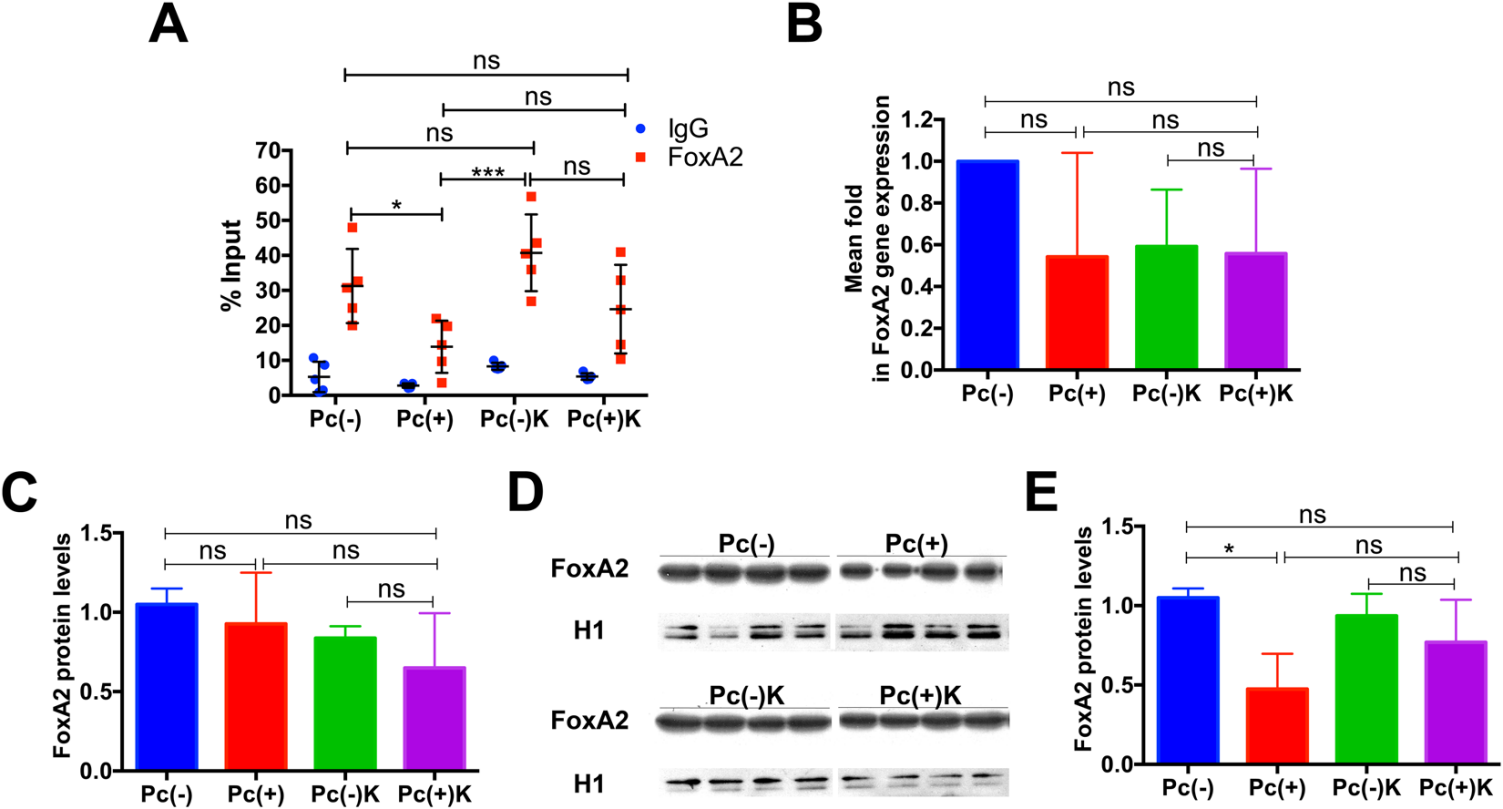
Binding of FoxA2 to Muc5b promoter depends on STAT6 pathway. (**A**) Chromatin Immunoprecipitation (ChIP) method shows that specific binding of FoxA2 to Muc5b promoter (in gray squares) is less in *Pneumocystis*-infected rats compared to non-infected rats without or with Kaempferol (^*^P = 0.0159 and ^***^P = 0.0079). These significant decreases are lost with addition of Kaempferol to *Pneumocystis*-infected rats suggesting reversal. Controls (IgG) are shown in blue. Results are expressed as percentage of input. n = 5. (**B**) mRNA levels of FoxA2 are not significantly different in different experimental groups suggesting decrease in FoxA2 binding to Muc5b promoter is not influenced by gene expression. Results are expressed as mean fold change in gene expression using 2^−ddCt^ method and the group of non-infected, saline treated, rats (Pc−S) as reference (n = 4). (**C**) Plot of whole cell protein levels of FoxA2 in lung samples of each animal group as detected by WB (n = 4). Lack of differences suggests that changes in FoxA2 are compartmentalized within the cell. Results are indicated as mean +/− SD and were normalized by Actin. (**D**,**E**) Representative western blot of nuclear protein levels of FoxA2 in lung samples of each experimental group (n = 4) (**E**) suggesting a decrease in FoxA2 nuclear localization in infected rats that reverts with addition of Kaempferol as shown in (**F**) Plot of the WB shown in D. Results are expressed as mean +/− SD and were normalized by H1 levels. ^*^ indicates *P* = 0.0286. ns indicates no significance. Statistical comparisons were made using Mann-Whitney test.

JAK/STAT6 pathway normally regulates mucin transcription through the repressor FoxA2 which binds to the respective mucin promoter to control transcription and consequently mucin mRNA levels. A highly conserved FoxA2 binding site was identified by comparison of DNA sequences of the human, mouse and rat Muc5b promoter regions. The presence of FoxA2 in this Muc5b promoter region was first confirmed using chromatin immunoprecipitation (ChIP). Binding of FoxA2 decreased in 0,5-fold in *Pneumocystis*-infected animals compared to controls (Pc− v/s Pc+) (Fig. 6A). Kaempferol partly reversed the effect of *Pneumocystis* (Pc+v/s Pc + K). No differences were detected in FoxA2 mRNA and protein levels analyzed from total RNA and protein extracts, after Kaempferol treatment (Fig. 6B,C). However, a significant decrease in FoxA2 nuclear protein levels was detected in animals infected with *Pneumocystis* receiving saline only (Fig. 6D,E). This effect of *Pneumocystis* in lowering nuclei FoxA2 levels was attenuated completely by Kaempferol (Fig. 6D,E).

## Discussion

This work documents increased expression of MUC5B associated to *Pneumocystis* primary infection in autopsy lung samples from immunocompetent infants dying in the community (Fig. 1), and the progression of gel-forming mucins (Muc5b and Muc5ac), and potential for pharmacological reduction of mucus through the STAT6/FoxA2 pathway, in an animal model of primary infection by *Pneumocystis*.

We have previously documented increased MUC5AC associated to *Pneumocystis* in infant lung autopsy samples and that this increase was unrelated to common respiratory viruses^19,24^. This association has proven consistent^19,24^. This work ads to show that *Pneumocystis* strongly stimulates both main gel-forming mucins suggesting an altered mucus physiology during the primary infection^1,19^. Overproduction of MUC5B has also been associated with chronic obstructive pulmonary disease (COPD), and to the development of interstitial pulmonary fibrosis (IPF) in adults, and overproduction of MUC5AC with asthma^2^. The role and duration of *Pneumocystis* associated mucus changes in these medical conditions associated to *Pneumocystis*, remain to be determined.

Muc5b levels increased 10 to 15 times, and this increase occurred earlier than that of Muc5ac in animals in this study suggesting a faster Muc5b than Muc5ac response during *Pneumocystis* primary infection (Fig. 2A–D), and that Muc5b may play a defensive role against *Pneumocystis* as recently described for bacterial pathogens^8^. Of interest, secreted MUC5AC increased later in the infection course (Fig. 2A,B). This progression is consistent with the findings that the primary infection by *Pneumocystis* can drive the development and progression of asymptomatic asthmatic-type airway pathology as described^25,26^, and is also consistent with the role of Muc5ac in the development of airway hyperreactivity^7^ adding to the possibility of altered airway responses during the course of this primary infection^19,26^. Muc5B was documented histologically in bronchi but not in bronchioles (Fig. 3). Goblet, mucus producing, cells are abundant in bronchi and rare in bronchioles and findings suggest stimulation of existing goblet cells rather than metaplasia during the time course of *Pneumocystis* infection evaluated in these experiments. This animal model reproduces the natural route of contagion and progression of this fungal infection in humans^32^, and documents an ongoing immune response throughout the course of the *Pneumocystis* primary infection^26^. Translating these findings to the human situation suggests that *Pneumocystis* may prime the lung to altered responses to co-infecting respiratory pathogens and support the hypothesis that subclinical, unnoticed, *Pneumocystis* may contribute to the higher incidence of respiratory morbidity and increased rate of hospitalizations for respiratory cause that occurs in infants between 2 and 5 months of age^22,23^. Respiratory viruses affect infants at any age. *Pneumocystis* however, is highly frequent between 2 and 5 months of age and is rare after the age of 6 months^18–21^. This possible, yet-unproven, role as a sub-clinical fungal co-pathogen is suggested by the extremely high prevalence of *Pneumocystis* in infants^19,21^, that peaks coincidently at this age window^18–21^ and by the pulmonary pathology documented in animal models^25–28^.

An important mechanistic result of this work is the documentation that Muc5b production is to certain extent dependent on STAT6/FoxA2 pathway and that pharmacological intervention of the STAT6/FOXA2 pathway, as has been previously proposed^33^, can serve as a mechanism for modulation of mucus during *Pneumocystis* infection. STAT6 is an important pathway in the innate immune response to *Pneumocystis* as suggested in our previous studies related to the association of *Pneumocystis* and MUC5AC increase in infant lungs and documented in STAT6 knockout mice^24,27^. STAT6 is activated by IL13 that favors mucin expression through binding inhibition of transcriptional repressor FoxA2 to the mucin promoters^9,34^. FoxA2 reduces the expression of mucins and, therefore, binding-inhibition of FOXA2 is necessary to induce enhanced expression of MUC5B^35^. Paradoxically, increased methylation of FoxA2 binding site in the mucin promoter has been shown to enhance overexpression of MUC5B^14^, indicating that regulation mucins expression is complex. Kaempferol, was selected as a drug to reverse the FoxA2 binding-inhibition of *Pneumocystis* because kaempferol is a specific inhibitor of JAK3, which phosphorylates and activates STAT6^5,10,30^. We first documented increasing P-STAT6 levels by *Pneumocystis* activation and reversal by Kaempferol (Fig. 4), and then, the stimulatory effect of *Pneumocystis* in Muc5b secretion and reversal by Kaempferol (Fig. 5). However, the limited potential for Muc5b pharmacological modulation via the STAT6/FoxA2 pathway was confirmed in these animals by the partial reversal of the binding of FoxA2 to Muc5b promoter associated to *Pneumocystis* by the addition of Kaempferol (Fig. 6). Therefore, documenting that *Pneumocystis* stimulation of Muc5b via the STAT6/FoxA2 pathway, occurs by inhibition of the FoxA2 repressor and, can be partly reverted by Kaempferol. Other potential pharmacological intervention that has been suggested to modulate the mucus hypersecretion associated to *Pneumocystis* in infants and patients with COPD, is to increase methylation of histone 3 which is known to suppress STAT6^35^ suggesting epigenetic modifications also deserve exploration^35,36^. Modulation of mucus is complex and additional mucogenic pathways like EGFR and NFkB may be involved. For example, STAT6 and NFkB pathways that may interact via transcription factors P-STAT6 and NFkB subunits p50 and p65^37^ or through a non-canonical pathway where IL13 stimulation has been documented to activate NFkB^38^.

In summary, this work documents a relevant mucus response to *Pneumocystis* primary infection in the immunocompetent host. The progression of individual gel forming mucins over time showed an earlier and higher increase of Muc5b as part of the innate defensive response, and a slower increase of Muc5ac that suggests an ongoing Th2-type immune response that is more related to airway sensitization against this fungus occurring during the primary infection. *Pneumocystis* sensitizes the airway to strikingly potent constrictive responses^39^. Results show also, that the STAT6/FOXA2 pathway is involved in the positive regulation of mucus during *Pneumocystis* primary infection, and that pharmacologic modulation of this pathway may be a suitable strategy to limit *Pneumocystis*-induced mucus overproduction.

## Methods

### Infant lung samples

Infant autopsies were legally required as per Chilean law for all infants dying in the community, and conducted at the Servicio Médico Legal de Santiago, which is the coroner’s office institution for the Metropolitan Area of Chile. Autopsy lung samples from infants without an ascertainable cause of death and categorized as Sudden Unexpected Infant Deaths (SUID) were selected using a randomization protocol available at www.randomizer.org from a pool of 39 *Pneumocystis*-positive and 20 *Pneumocystis*-negative samples stored at -80 °C in our laboratory without possible identifiers to link their identity^19^. Samples were numbered 1 to 39 in *Pneumocystis*-positive group and 1 to 20 in *Pneumocystis*-negative group and eight positive and eight negative samples were selected.

### Rat model of *Pneumocystis* Primary Infection

Sprague Dawley rats from same colony were used for each individual experiment. Timed-pregnant rats were randomly assigned to be exposed or non-exposed to *Pneumocystis* as described^26^ and received tylosin tartrate (93 mg/l) during 3 weeks prior to delivery to prevent bacterial infections. Control pregnant rats received cotrimoxazole (TMS) in addition to secure prevention of *Pneumocystis*. Dams and pups of the exposed groups were co-housed at birth with seeding rats with *Pneumocystis* pneumonia as described^26^. Female pups were selected at weaning. However, the effect of gender in *Pneumocystis* primary infection has not been studied. They were sacrificed at 45, 60 and 75 days from birth under deep anesthesia with Xylazine 10 mg/kg and Ketamine 100 mg/kg. Half of animals at each sacrifice were exsanguinated and their lungs kept at -80 °C until processed to extract RNA and proteins. The other half were perfused via cava vein with 3.7% PBS-buffered formalin and removed after 24 hours as described^26^.

### Administration and evaluation of Kaempferol effect

The same model^26^ was reproduced to evaluate pharmacologic modulation of STAT6/FoxA2 pathway with Kaempferol at day 70. *Pneumocystis*-positive and *Pneumocystis*-negative rat groups were each separated in two groups to start Kaempferol 7 mg/Kg or saline daily intra-peritoneal injections for two weeks from days 55–70. All animals were sacrificed at day 70 for mucus and STAT6/FoxA2 pathway markers determinations.

### Detection and quantification of *Pneumocystis*

Total genomic DNA was isolated from fresh-frozen homogenized lung tissue 0.3 g aliquots using QIAmp DNA mini kit (Qiagen). *Pneumocystis* was identified by nPCR using *P*. *carinii* specific oligonucleotide primers as described^40^. *Pneumocystis* burden was determined by quantification *dhfr* gene using qPCR with primers as described^26^. Copies of the *dhfr* gene were determined by interpolation in a calibration curve. PCR reactions conditions utilized an initial denaturation step of 5 minutes at 94 °C, 45 cycles of 20 seconds at 94 °C, 20 seconds at 57 °C, and 20 seconds at 72 °C.

### Mucus determinations

Paraffin embedded lungs sections (3 μm) were stained with Alcian Blue (1% AB in 3% glacial acetic acid, pH = 2.5) and periodic acid-Schiff’s reagent (AB/PAS) using standard protocols. All images were acquired using an Olympus BX60 microscope connected to a Q-IMAGING Micropublisher 3.3 RTV camera (QImaging, Burnaby, BC, Canada). Immunohistochemical staining was done as per manufacturer protocols sc-2018 using anti-MUC5B primary antibody sc-135508 (Santa Cruz Biotechnology, USA) and anti-Muc5ac ab 64259 kit (Abcam, UK). Muc5ac and Muc5b immunohistochemical quantifications were done by estimating the percentage of airway epithelium area stained by each respective mucin antibody using Image Pro Plus software version 5.1.0 (Media Cybernetics Inc., Rockville, MD, USA) in an average of 16 optical fields (3–35 range) per animal using a 10x objective (100x magnification). Each mucin stained area per animal represents the mean percent airway epithelial stained area value of the individual airways measured in that animal. Means per animal were then used to calculate the group mean. Airways were classified according to diameter in bronchi (>250 μm), bronchioles (50 to 250 μm) and terminal bronchioles (<50 μm)^31^.

### Muc5ac and Muc5b qRT-PCR determinations in rat lungs

Lungs were placed in RNA later (QIAGEN, USA) immediately after sacrifice, kept for one night at 4 °C and then frozen at -80 °C until total RNA extraction. Total RNA extraction was performed according QIAGEN RNeasy mini spin columns method, starting with 30 mg of tissue. Total RNA (2 μg) was reversely transcribed into cDNA using random hexamer primers and SuperScript IV (Invitrogen, USA). The resulting one-stranded cDNA was diluted 1:2 and 1 μl used for PCR reactions. Amplification of Muc5b and Muc5ac was performed using the SensiMix SYBR Hi-ROX Kit (Bioline, UK) using a RotorGene 6000 Series equipment (Corbett Research, Canada). Primer sets used for amplification were: Muc5b F: 5′ CCTGAAGTCTTCCCCAGCAG 3′; Muc5b R: 5′ GCATAGAATTGGCAGCCAGC 3′; Muc5ac F 5′ ACCACGGATATCAGAACCAGC 3′; Muc5ac R 5′ TGTCAAGCCACTTGGTCCAG 3′. PCR reactions were performed with the following conditions: Initial denaturation of 5 minutes at 94 °C, 45 cycles of 30 seconds at 94 °C, 20 seconds at 60 °C, 20 seconds at 72 °C. Actin was used as internal amplification control^26^. mRNA quantifications were normalized by actin. Results were compared using the corresponding control determination at each sacrifice point as the reference value.

### Western blotting determinations in human and rat lungs

30 mg samples from each lung were homogenized in 1 ml of standard RIPA (50 mM Tris pH 7.4, 1% NP-40, 0.5% sodium deoxycholate, 150 mM NaCl, 1 mM EDTA) buffer supplemented with protease inhibitor cocktail (Roche, Germany) and sodium dodecyl sulfate (0.01% final concentration). Samples were centrifuged twice to eliminate cellular debris and lipids after 30 minutes of ice incubation. 30 μg per sample were analyzed for MUC5B, total STAT6, phosphorylated STAT6 (P-STAT6) and FoxA2 by immunoblotting. Proteins were separated by 8% polyacrylamide denaturing gel and transferred to PVDF membrane (ThermoScientific, USA). Membranes were blocked with 5% non-fat milk in TBS for one hour at room temperature and then probed with anti-MUC5B (sc-135508, Santa Cruz Biotechnology, USA), anti-STAT6 (sc-621, Santa Cruz Biotechnology, USA), anti-P-STAT6 (sc-11762, Santa Cruz Biotechnology, USA), anti-FoxA2 (sc-6554, Santa Cruz Biotechnology, USA) or anti-actin (sc-1616, Santa Cruz Biotechnology, USA) diluted 1/2000 in 1% non-fat milk in TBS during one night at 4 °C. Complexes were visualized by chemiluminescence using the ECL Detection System and X-ray films (ThermoScientific, USA). Bands were quantified using ImageJ software and normalized against actin (NIH, USA). (All original gels are provided as supplementary material). Results were compared using the corresponding control animal determination as the reference value.

### Nuclear protein extractions in rat lungs

200 mg of fresh lungs were homogenized in 1,5 ml of Buffer A (10 mM HEPES pH 7.9, 10 mM KCl, 1.5 mM MgCl_2_, 0.6% NP-40, complete protease inhibitor cocktail) and centrifuged at 350 g, 30 seconds at 4 °C, to eliminate debris. Supernatants were put in ice for 5 minutes and then centrifuged during 5 minutes at 6000 g. Pelleted materials were suspended in 200 μl of Buffer B (10 mM HEPES pH 7.9, 10 mM KCl, 1.5 mM MgCl_2_, 1.2 M Sucrose, 0.5 mM DTT, complete protease inhibitor cocktail) and then centrifuged at 13000 g during 30 minutes at 4 °C. Supernatants were discarded, and pellets resuspended in 100 μl of Buffer C (20 mM HEPES pH 7.9, 420 mM NaCl, 1.5 mM MgCl_2_, 0.2 mM EDTA, 0.5 mM DTT, 25% glycerol, complete protease inhibitor cocktail). Samples were incubated in ice for 20 minutes. Nuclear debris were eliminated by 2 minutes centrifugation at 6000 g at 4 °C. Nuclear proteins were quantified in supernatants and stored at -80 °C. 10 μg of each sample were analyzed to detect FoxA2 and H1 as internal control (sc-393358, Santa Cruz Biotechnology, USA)^41^.

### Chromatin immunoprecipitation in rat lungs

30 mg aliquots of fresh lungs were homogenized in 300 μl of iced-PBS in 1% formaldehyde final concentration as described^42^. Chromatins were incubated with 2 μg of anti-FoxA2 (sc-6554, Santa Cruz Biotechnology, USA) or of IgG (sc-2027, Santa Cruz Biotechnology, USA). Precipitated DNA was collected using QIAquick^®^ PCR purification kit (QIAGEN, USA). DNA was suspended to a final volume of 50 μl. Aliquots of 3 μl were analyzed by qPCR to amplify FoxA2-site in the rat Muc5b promoter. Primers used were: PMuc5bF: 5′ CTTCCTTCTCCATGGCCTC 3′, PMuc5bR: 5′ CCATCCCTCCAATTCCTGC 3′. PCR reactions were performed with the following conditions: Initial denaturation of 5 minutes at 94 °C, 45 cycles of 30 seconds at 94 °C, 20 seconds at 60 °C, 20 seconds at 72 °C. Amplification product included the FoxA2 binding site, TATA-box, transcription start and the first 11 codons of the protein.

### Statistics

Sample size for animal experiments was estimated considering a minimum of 60% difference in means per group with 95% potency for all comparisons using online tool available at http://oep.umh.es/calculo-del-tamano-muestral/. GraphPad Prism 6.0 software (San Diego, CA, USA) was used to analyze the means ± Standard Deviations (SD) of qRT-PCR and immunoblotting determinations by non-parametric Mann-Whitney test, assuming non-normal distribution and give sample size < 10 individual determinations. Two-way ANOVA was used for comparisons in sequential determinations of mucins by qPCR and protein levels by western blot, with multiple comparisons using Bonferroni analysis. Differences were considered significant when *P* < 0.05. Original data is provided as supplementary material.

### Ethics approvals

The Ethics Commission for Studies in Human Subjects of the University of Chile School of Medicine approved this study under protocol CEISH #092-2013. Informed consent was not obtained because this was a retrospective study of stored samples without identifiers as to the subject of origin, which is in line with national laws and regulations. All methods were performed in accordance with the principles of the Declaration of Helsinki for medical research involving human subjects and with the relevant local guidelines and regulations.

The Bioethics Committee University of Chile School of Medicine approved the animal studies under protocol CBA0606 and experiments conducted in accordance with the Animal Protection Law of Chile (Law 20.380), and the Guide for the Care and Use of Laboratory Animals (8th Edition, National Academies Press, Washington DC).

## Supplementary information

Original WB images

Data for each figure

## Data Availability

The data that support the findings of this study are available from the corresponding author upon request.
