# Supplementary material for: Increase in secreted airway mucins and partial Muc5b STAT6/FoxA2 regulation during Pneumocystis primary infection
Source: Sci Rep. 2019 Feb 14;9:2078. doi: 10.1038/s41598-019-39079-4 (PMC6376022; doi:10.1038/s41598-019-39079-4)
Supplement: Supplementary file 1 — Original WB images [file 41598_2019_39079_MOESM1_ESM.docx]

**Tit****le**

**Increase in secreted airway mucins and partial Muc5b STAT6/FoxA2 regulation during *Pneumocystis* primary infection.**

**Author list and affiliations**

Diego A. Rojas, PhD^1^

Pablo A. Iturra, PhD^1^

Andrea Méndez, PhD(c)^1^

Carolina A. Ponce, MSc^1^

Rebeca Bustamante, BA^1^

Miriam Gallo, MD^2^

Pamela Bórquez, MD^2^

*Sergio L. Vargas, MD^1^

**^1^**Biomedical Sciences Institute, University of Chile School of Medicine, Independencia 1027, Independencia, Santiago 8380453, Chile. ^2^ Servicio Médico Legal de Santiago, Av. La Paz 1012, Independencia, Santiago 8380454, Chile.

*Correspondence to [svargas@med.uchile.cl]


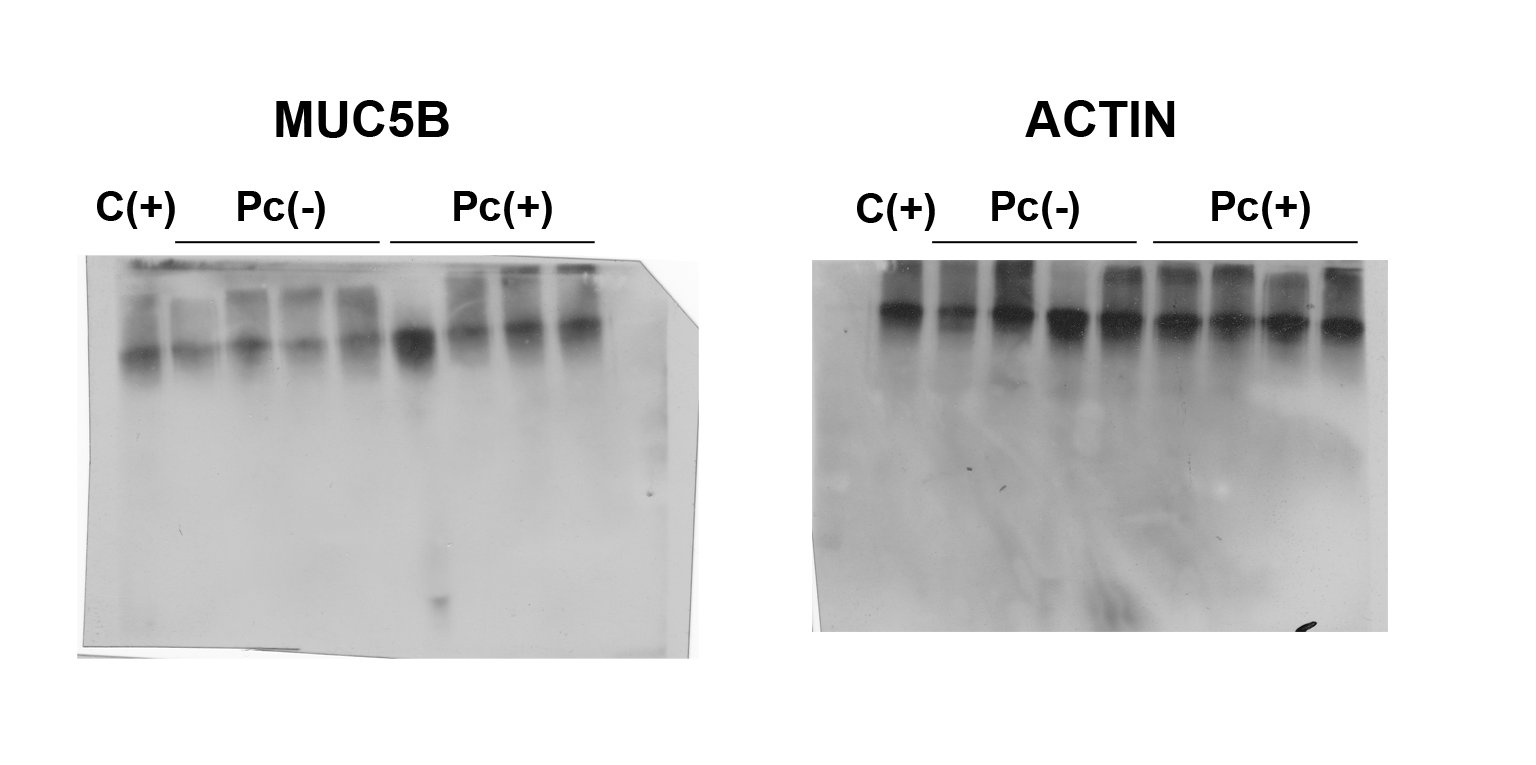


**Figure S1.** **Originals films of western blot presented in Figure 1.** The left panel shows protein levels of MUC5B and the right panel shows levels of actin. These analyses were performed using lung protein samples from *Pneumocystis* negative [Pc(-)] and positive [Pc(+)] autopsied infants. C(+) is a positive control sample from rat stomach in both films and is not included in the manuscript figure. Films are presented in gray scale.


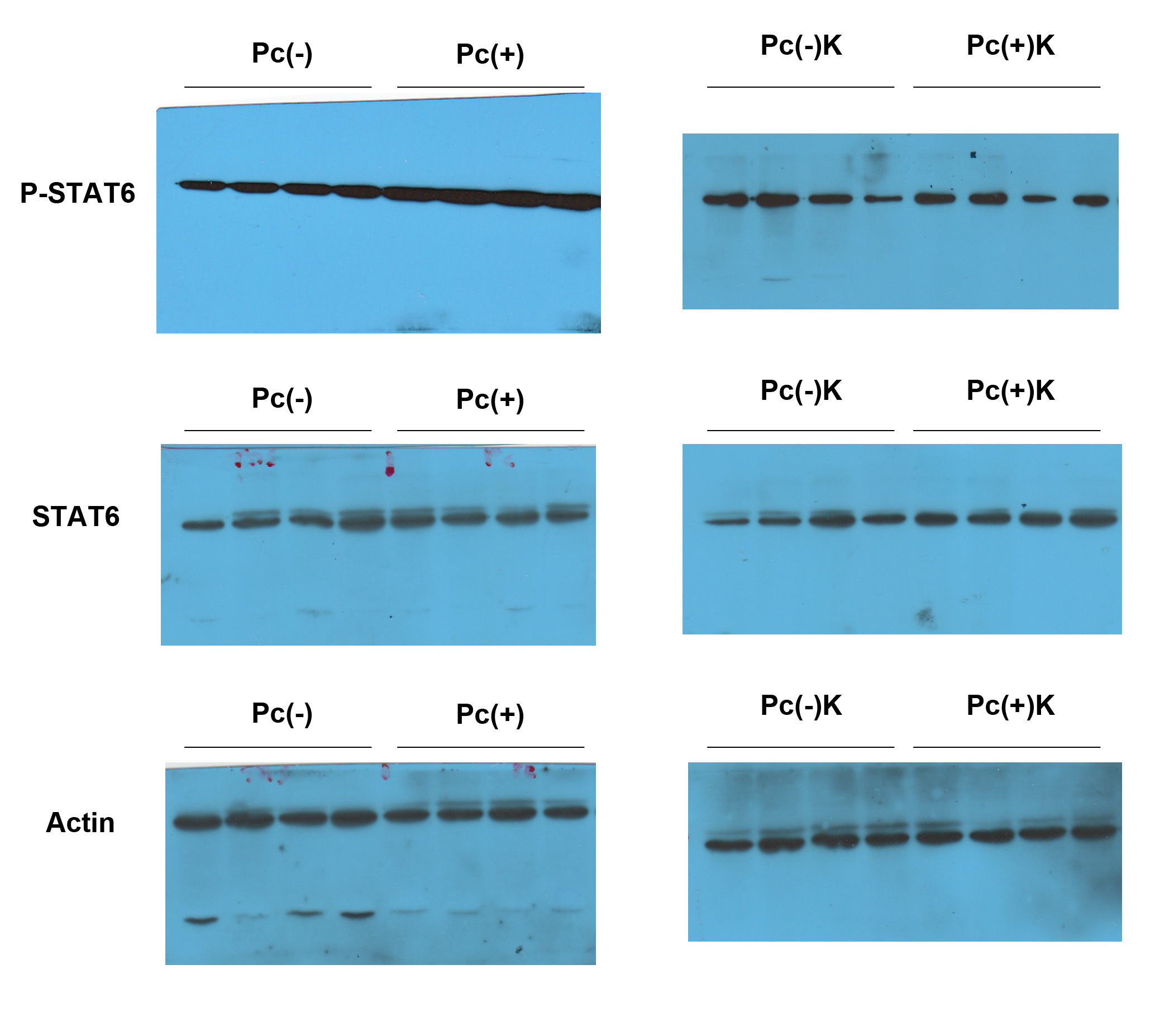


**Figure S2.** **Originals films of western blot presented in Figure 4.** Phosphorylated STAT6 (P-STAT6), total STAT6 and actin as sought by western blot of lung protein samples from the four experimental groups: *Pneumocystis*-negative [Pc(-)], *Pneumocystis*-positive [Pc(+)], *Pneumocystis*-negative treated with kaempferol [Pc(-)K] and *Pneumocystis*-positive treated with kaempferol [Pc(+)K]. The differences in the height of each figure is due to the different heights of the films used in the detection of the protein levels during chemiluminescent analysis. In addition, differences in the position of the detected protein bands in the films are due to differences in the time running of each SDS-PAGE.


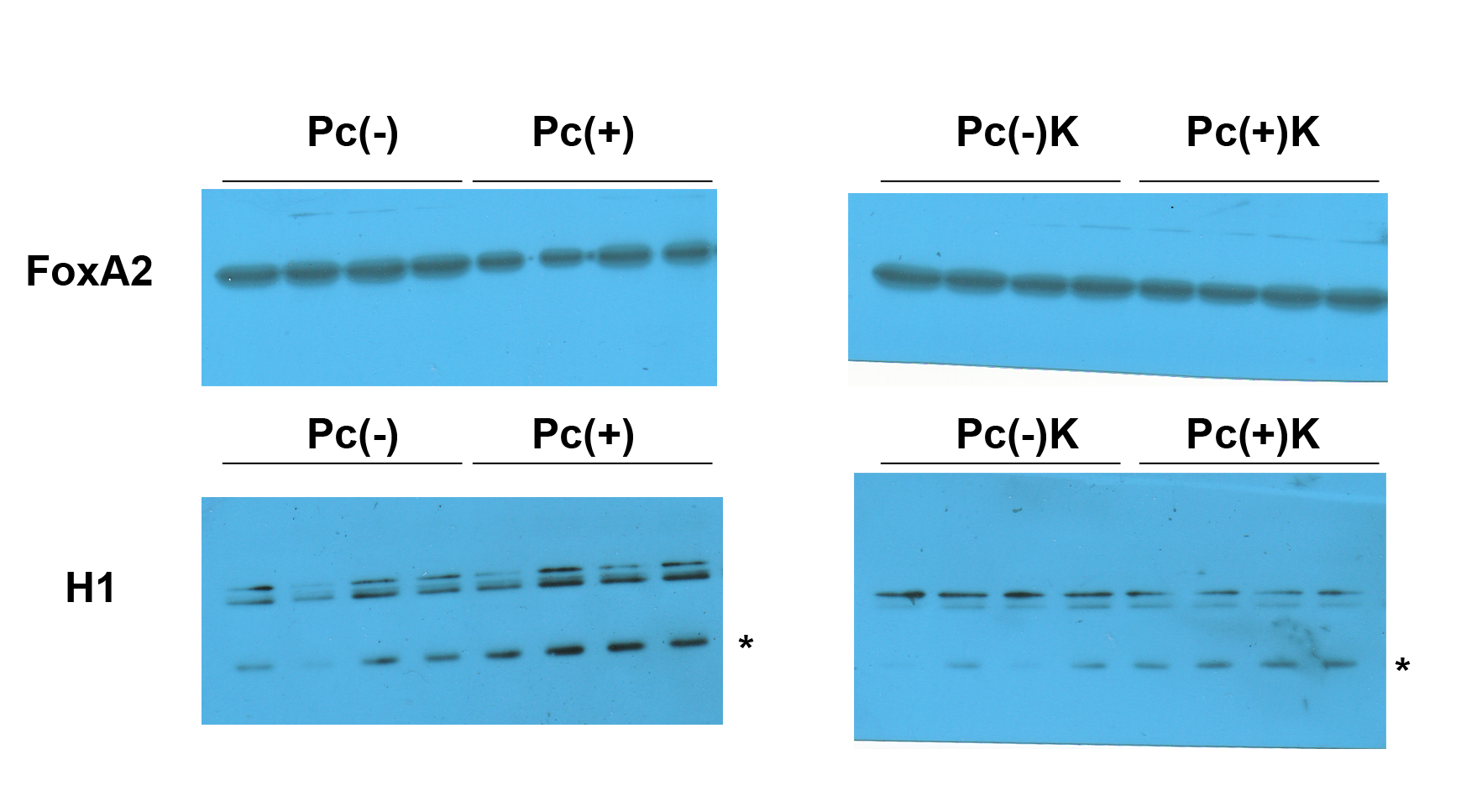


**Figure S3.** **Originals films of western blot presented in Figure 6.** FoxA2 and Histone 1 (H1) protein levels as sought by western blot using lung nuclear extract samples from the four experimental groups: *Pneumocystis*-negative [Pc(-)], *Pneumocystis*-positive [Pc(+)], *Pneumocystis*-negative treated with kaempferol [Pc(-)K] and *Pneumocystis*-positive treated with kaempferol [Pc(+)K]. The differences in the height of each figure is due to the different height of the films used in the detection of the protein levels during chemiluminescent analysis. In addition, differences in the position of the detected protein bands in the films are due to differences in the time running of each SDS-PAGE. (*) indicates non-specific protein band.
